# Radiation-induced gliomas represent H3-/IDH-wild type pediatric gliomas with recurrent *PDGFRA* amplification and loss of *CDKN2A/B*

**DOI:** 10.1038/s41467-021-25708-y

**Published:** 2021-09-20

**Authors:** Maximilian Y. Deng, Dominik Sturm, Elke Pfaff, Martin Sill, Damian Stichel, Gnana Prakash Balasubramanian, Stephan Tippelt, Christof Kramm, Andrew M. Donson, Adam L. Green, Chris Jones, Jens Schittenhelm, Martin Ebinger, Martin U. Schuhmann, Barbara C. Jones, Cornelis M. van Tilburg, Andrea Wittmann, Andrey Golanov, Marina Ryzhova, Jonas Ecker, Till Milde, Olaf Witt, Felix Sahm, David Reuss, David Sumerauer, Josef Zamecnik, Andrey Korshunov, Andreas von Deimling, Stefan M. Pfister, David T. W. Jones

**Affiliations:** 1 Hopp Children’s Cancer Center Heidelberg (KiTZ), Heidelberg University Hospital and German Cancer Resarch Center (DKFZ), Heidelberg, Germany; 2 Division of Pediatric Glioma Research, German Cancer Research Center (DKFZ), Heidelberg, Germany; 3 Department of Pediatric Oncology, Hematology and Immunology, Heidelberg University Hospital, Heidelberg, Germany; 4 Division of Pediatric Neurooncology, German Cancer Research Center (DKFZ) and German Consortium for Translational Cancer Research (DKTK), Heidelberg, Germany; 5 Department of Neuropathology, Institute of Pathology, Heidelberg University Hospital, Heidelberg, Germany; 6 Clinical Cooperation Unit Neuropathology, German Cancer Research Center (DKFZ) and German Consortium for Translational Cancer Research (DKTK), Heidelberg, Germany; 7 Department of Pediatric Oncology and Hematology, Essen University Hospital, Essen, Germany; 8 Division of Pediatric Hematology and Oncology, University Medical Center Goettingen, Goettingen, Germany; 9 Morgan Adams Foundation Pediatric Brain Tumor Research Program, Department of Pediatrics, University of Colorado School of Medicine, Aurora, CO USA; 10 Division of Molecular Pathology and Division of Cancer Therapeutics, The Institute of Cancer Research, London, United Kingdom; 11 Department of Neuropathology, Institute of Pathology and Neuropathology and Comprehensive Cancer Center Tübingen-Stuttgart, Tübingen University Hospital, Tübingen, Germany; 12 Department of Pediatric Hematology/Oncology, Children’s University Hospital, Tübingen, Germany; 13 Department of Neurosurgery, Division of Pediatric Neurosurgery, Tübingen University Hospital, Tübingen, Germany; 14 Clinical Cooperation Unit Pediatric Oncology, German Cancer Research Center (DKFZ) and German Consortium for Translational Cancer Research (DKTK), Heidelberg, Germany; 15 Department of Neuropathology, NN Burdenko Neurosurgical Institute, Moscow, Russia; 16 Department of Pediatric Hematology and Oncology, Motol University Hospital, Charles University, Prague, Czech Republic; 17 Department of Pathology, Motol University Hospital, Charles University, Prague, Czech Republic

**Keywords:** Cancer genomics, CNS cancer

## Abstract

Long-term complications such as radiation-induced second malignancies occur in a subset of patients following radiation-therapy, particularly relevant in pediatric patients due to the long follow-up period in case of survival. Radiation-induced gliomas (RIGs) have been reported in patients after treatment with cranial irradiation for various primary malignancies such as acute lymphoblastic leukemia (ALL) and medulloblastoma (MB). We perform comprehensive (epi-) genetic and expression profiling of RIGs arising after cranial irradiation for MB (n = 23) and ALL (n = 9). Our study reveals a unifying molecular signature for the majority of RIGs, with recurrent *PDGFRA* amplification and loss of *CDKN2A/B* and an absence of somatic hotspot mutations in genes encoding histone 3 variants or *IDH1/2*, uncovering diagnostic markers and potentially actionable targets.

## Abstract (web summary)

Radiation-induced gliomas (RIGs) have been reported in patients after treatment with cranial irradiation for various primary malignancies but their origin are still unclear. Here, the authors define the genomic, epigenetic and transcriptional landscape of 32 RIGs cases.

## Introduction

Radiation therapy (RT) constitutes an essential element in the standard treatment of many cancers, improving clinical outcomes of patients with childhood malignancies including leukemia and central nervous system (CNS) tumors^1^. However, radiation-induced malignancies are observed post-RT in a subset of patients^2,3^, especially in pediatric patients due to their prolonged follow-up in case of long-term survival^4,5^.

Radiation-induced gliomas (RIGs) are known to arise in a subset of patients receiving cranial RT for the treatment of different primary malignancies including acute lymphoblastic leukemia (ALL) and medulloblastoma (MB), occurring after a variable latency ranging from 2.5 to 35 years after irradiation (Table 1)^6–18^.

**Table 1:** Summary of radiation-induced gliomas reported in previous studies.

Reference	Number of cases	Primary malignancy	Latency period (years)	Age at RIG diagnosis (years)	Gender ratio (m:f)	Death (y/number of cases)	OS (months)	*PDGFRA* status	*CDKN2A/B* status	Further genetic alterations
Brat et al.^6^	6	ALL, Pineal tumors, Lymphoblastic Lymphoma, Pituary adenoma, Rhabdomyosarkoma, Craniopharyngeoma	5–23	9–60	2:1	N.D.	N.D.	N.D.	N.D.	–
Donson et al.^7^	5	Burkitt’s Lymphoma, MB, low-grade astrocytoma, ependymoma, ALL	3–15	11–23	3:2	5/5	1–10	Overexpressed	N.D.	–
Lopez et al.^20^	12	Craniopharyngioma, Germinoma, Medulloblastoma, Pineocytoma, ALL, Hodgkin’s Lymphoma	4–41	7–48	8:4	5/7	<24	Amplified in 42%, mutated in one case	Loss in 33%	Amplified *CDK4* in 33%, amplified *MET* in 17%
Nakao et al.^13^	4	Pituary adenoma, Meduloblastoma, Craniopharyngioma, PNET	22–29	32–55	3:1	4/4	11–30	N.D.	N.D.	*IDH1* and *H3F3A* wild type
Paugh et al.^21^	10	ALL, Germinoma, MB, Ependymoma	N.A.	8–19	N.A.	7/9	8–91	Amplified in 50%	Loss in 50%	1q gain (50%), 1p loss (50%), 13q loss (70%)
Phi et al.^22^	5	Medulloblastoma (SHH, Group3)	4.3–10	9.2–17	2:3	N.D.	N.D	Missense mutation, gene fusion	N.D.	*TP53* (somatic mutations or 17p loss), 7q gain (*EZH2*)
Romeike et al.^16^	7	ALL, MB	7–14	9–19	4:3	7/7	9–27	N.D.	N.D	–
Walter et al.^18^	7	ALL	7–14	10–24	4:3	5/7	0.1–93	N.D.	N.D.	–

*OS* overall survival, *N.D.* not determined.

RIGs commonly display an aggressive clinical course associated with poor prognosis, making early and precise diagnosis (including distinguishing RIGs from recurrences of the primary tumor) crucial for optimal treatment planning^7,8^. This is particularly challenging in cases where putative recurrent CNS tumors are not biopsied or resected, and where material for histological or molecular confirmation is therefore lacking.

Histopathologically, most RIGs present as high-grade gliomas (HGG) reminiscent of their sporadic counterparts^7,8,13,14,19^, and histopathological features to distinguishing them from de-novo HGG are missing^6,7^. A report of morphological features of both MB and HGG within secondary tumors led to the hypothesis of transformation from MB (stem-)cells as a possible route for RIG development^11^, while the higher genetic homogeneity of RIGs compared to de-novo pediatric HGG is suggestive of a common origin for RIGs^7^. Genetic alterations including gain of chromosome arm 1q, loss of *CDKN2A/B*, and *PDGFRA* amplification or overexpression have been frequently observed in RIGs^13,20–22^, and—in contrast to sporadic pediatric HGG—recurrent somatic hotspot mutations in genes encoding histone 3 variants or *IDH1/2* were found to be uncommon^13,20–22^. To date, however, most series have been relatively small, and there remains some lack of clarity about the origin of RIGs and the underlying molecular and biological mechanisms leading to their formation.

Recent studies have shown that DNA methylation profiling represents a robust and reproducible approach to classify CNS tumors into clinically meaningful entities^23–26^. Thereby, pediatric HGG represent a heterogeneous group of CNS tumors, clearly distinguishable from their adult counterparts^21,27–29^, that can be classified into distinct subgroups with characteristic genetic and epigenetic alterations and clinical associations^30–33^. Next-generation sequencing and transcriptomic profiling approaches represent complementary tools for molecular characterization of tumorigenic processes.

Here, we performed comprehensive molecular characterization of RIGs to detect distinctive, (epi-)genetic features which might predict or explain their formation after RT, and/or which may act as diagnostic or therapeutic markers.

## Results

### Clinical characteristics

**Fig. 1:**
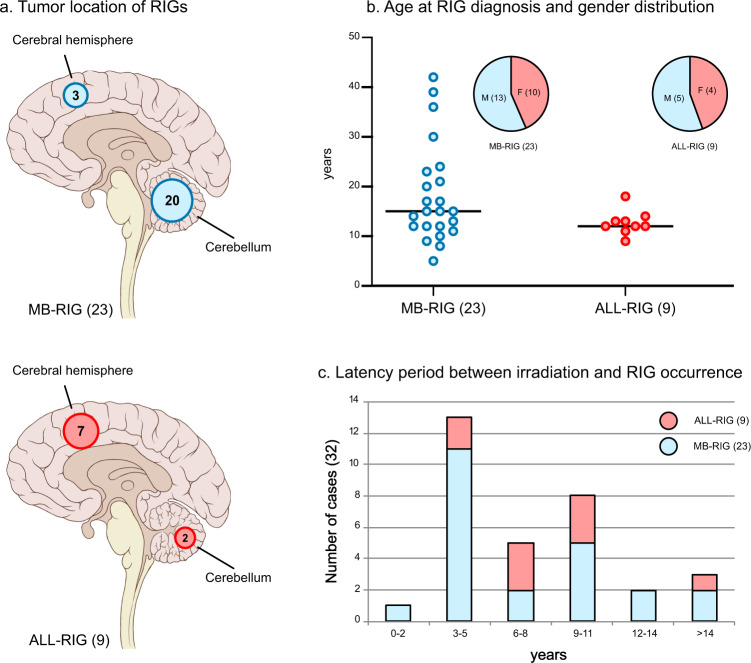
Clinical patient characteristics. Tumor location for 23 MB-RIG and 9 ALL-RIG illustrated with circled numbers indicating the number of patients with RIG occurrence in the respective brain region (**a**). Significant differences in tumor location were observed between MB-RIG and ALL-RIG patients (*p* < 0.01). Gender distributions, age at RIG diagnosis (**b**) and latency period (**c**) between cranial irradiation and RIG diagnosis are shown with numbers in brackets indicating group size. MB-RIG patients displayed a wider distribution regarding the age at RIG diagnosis and latency period. RIG radiation-induced glioma, MB-RIG post-medulloblastoma RIG, ALL-RIG post-ALL RIG.

Patients in our series developing a radiation-induced glioma (RIG, *n* = 32) were previously treated with cranial irradiation for MB (MB-RIG, *n* = 23) or ALL (ALL-RIG, *n* = 9). A variety of different RT regimens was administered, including craniospinal radiotherapy in MB patients or prophylactic cranial irradiation in ALL patients. In line with a higher probability of RIG occurrence in areas receiving a higher effective radiation dose, most MB-RIG tumors were encountered in the cerebellum (20/23, 87% vs. 3/23 in the cerebral hemisphere), the location with the highest dose in RT. ALL-RIG tumors predominantly appeared in the cerebral hemisphere (7/9, 78%, vs 3/23 in MB-RIGs, *p* < 0.01; Fig.1a), with only two cases arising in the posterior fossa. The gender ratio was slightly shifted towards males (male:female ratio: 1.28:1) (Fig. 1b). The latency for RIG occurrence ranged from 2 to 30 years in MB patients (*n* = 23, median: 5 years) and from 3–17 years in ALL patients (*n* = 9, median: 8 years; Fig. 1c).

**Fig. 2:**
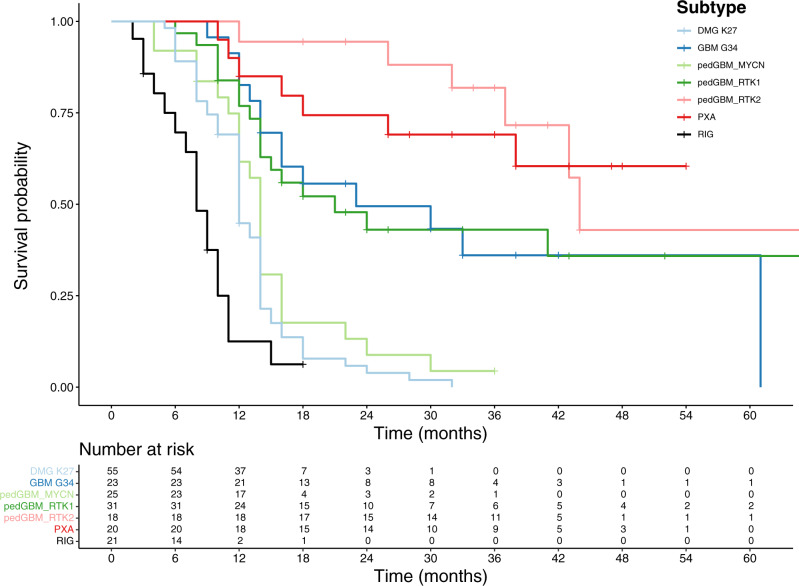
Kaplan–Meier curves illustrating overall survival for RIGs with representative CNS tumor entities. PXA pleiomorphic xanthoastrocytoma, pedGBM_RTK1 pediatric glioblastoma subclass RTK1, pedGBM_RTK2 pediatric glioblastoma subclass RTK2, pedGBM_MYCN pediatric glioblastoma subclass MYCN, DMG K27 diffuse midline glioma H3 K27 mutant, GBM G34 glioblastoma, H3.3 G34 mutant.

For 16/23 MB-RIG and 5/9 ALL-RIG patients with available follow-up data, we observed an aggressive clinical course that was as bad as or even slightly worse than histone 3 K27M-mutant tumors – with 13 out of 16 MB-RIG and four out of five ALL-RIG patients showing fatal outcome during the follow-up period (OS: 6 months, range: 3–10 months vs 8.5 months, range: 2–18 months in MB-RIG patients; Suppl. Table 1). One ALL-RIG (RIG_09, follow-up period: 3 months) and 3 MB-RIG patients diagnosed at the age of 14 year (RIG_22, follow-up period: 9 months), 15 years (RIG_30, follow-up period: 3 months), and 39 years (RIG_14, follow-up period: 18 months) were known to be alive at last follow-up, with the latter showing signs of progression after 12 months (Fig. 2).

### DNA methylation profiling of radiation-induced gliomas and primary medulloblastomas

**Fig. 3:**
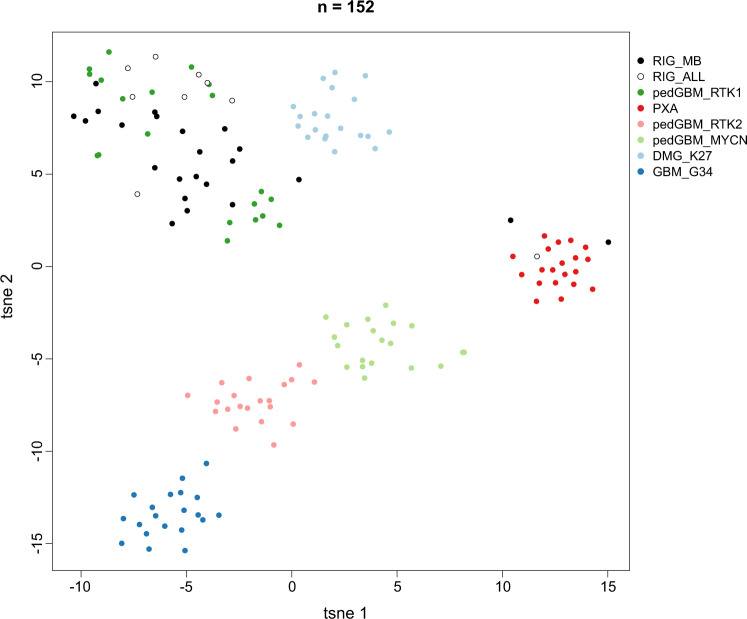
Molecular classification of RIGs by DNA methylation profiling. Unsupervised analysis of 32 RIG tumors was conducted based on the 10,000 most variably methylated probes. Through a t-SNE analysis, the majority of RIG tumors form a homogenous group of tumors resembling the pediatric GBM RTK1 group. ALL-RIG and MB-RIG samples were compared with 120 well-characterized reference samples representing CNS tumors of known histological and/or molecular subtype. PXA pleiomorphic xanthoastrocytoma, pedGBM_RTK1 pediatric glioblastoma subclass RTK1, pedGBM_RTK2 pediatric glioblastoma subclass RTK2, pedGBM_MYCN pediatric glioblastoma subclass MYCN, DMG_K27 diffuse midline glioma H3 K27 mutant, GBM_G34 glioblastoma, H3.3 G34 mutant.

DNA methylation patterns of both MB- and ALL-RIGs were analyzed by unsupervised clustering and t-SNE alongside 120 reference samples from pediatric HGG subgroups with known molecular features. The majority of RIGs after both primary indications closely resembled those of pedGBM_RTK1 tumors (29/32, 91%; Fig. 3a, b)^32^. Two MB-RIG and one ALL-RIG showed a methylation pattern more similar to pleomorphic xanthoastrocytomas (PXA) (Fig. 3b). Thus, RIG DNA methylation patterns suggest a high degree of similarity (and likely common origins) between tumors, and were clearly distinct from any other HGG DNA methylation class.

**Fig. 4:**
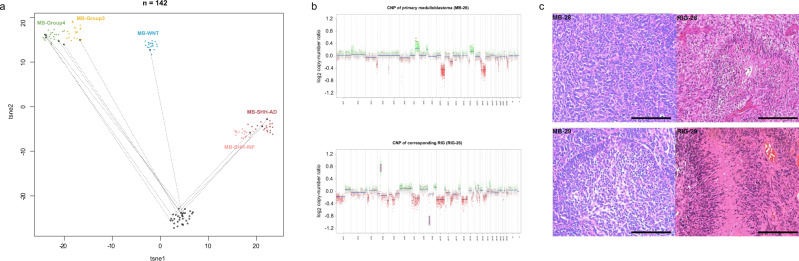
Comparison of matched primary MB and post-radiation glioma samples. T-SNE analysis of primary MB and post-MB RIG compared with existing MB subgroups (**a**) and representative copy-number profile (**b**) reveal distinct genome-wide methylation patterns and recurrent high-level *PDGFRA* amplification and *CDKN2A/B* deletion. Available primary MB and post-MB RIG samples (*n* = 11 pairs) were investigated with 100 MB reference samples representing established MB subtypes^57,70^. MB and RIG pairs from our series were encircled and linked with dotted lines. Histological staining for hematoxylin and eosin (**c**) of two paired primary medulloblastoma and radiation-induced gliomas (MB/RIG_28, MB/RIG_29) display distinct morphological characteristics. Scale bar represents 200 μm. T-SNE t-stochastic neighboring embedding, MB medulloblastoma, MB-SHH-INF medulloblastoma sonic-hedgehog infant subgroup, MB-SHH-AD medulloblastoma sonic-hedgehog adult subgroup, CNP copy-number profile.

The 11 pre-RIG primary MBs for which DNA was available were also profiled, and found to represent all four major MB subgroups (MB-WNT: 1/11; MB-SHH: 3/11; MB-Group 3: 2/11, MB-Group 4: 5/11), with no obvious enrichment for a particular primary subtype relative to their overall incidence rate. DNA methylation and CNV profiling confirmed the distinct biological nature between the primary MB and secondary RIG using t-SNE and copy-number analyses (Fig. 4a, b). Histological staining for hematoxylin and eosin (Fig. 4c) of two paired primary medulloblastoma and radiation-induced glioma couples (MB/RIG_28, MB/RIG_29) display distinct morphological characteristics.

### RIGs display recurrent *PDGFRA* amplification and loss of *CDKN2A/B*

**Fig. 5:**
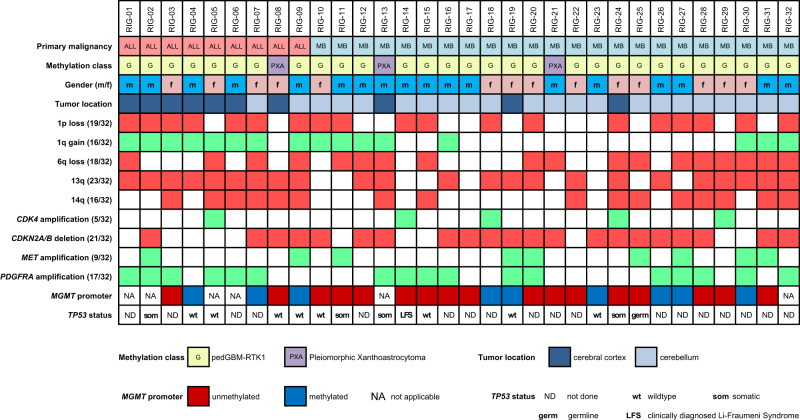
Oncoplot illustrating the genomic landscape in RIGs. Summary of recurrent, characteristic genetic alterations (focal copy-number alterations, mutations) identified through DNA methylation arrays and whole-exome/gene panel sequencing. *MGMT* O6-methylguanine–DNA methyltransferase. ALL acute lymphoblastic leukemia, MB medulloblastoma.

Amplification of *PDGFRA* (6/9 ALL-RIG; 11/23 MB-RIG) and loss of *CDKN2A/B* (4/9 ALL-RIG; 17/23 MB-RIG) represented the most common copy-number alterations (CNAs) in RIGs, with 2/8 ALL-RIG and 8/23 MB-RIG patients exhibiting co-occurrence of both (Fig. 5). This is in keeping with the methylation profile of these tumors matching to the pedGBM_RTK1 group, where both changes are also well-known characteristic features. Indeed, the overall frequency of *PDGFRA* amplification in the RIG cohort (17/32, 55%) is higher than in an unselected pedHGG cohort, but is not significantly higher than that reported in the pedGBM_RTK1 subgroup (33%, *p* = 0.14)^32^. Amplification of *MET* (9/32, 28%; previously also linked to *MET* fusion in pediatric GBMs^34^) and *CDK4* (5/32, 16%) were frequently encountered in our cohort. Additional broader chromosomal CNAs included gain of chromosome arm 1q (16/32, 50%), 1p deletion (19/32, 59%), 6q deletion (18/32, 56%), 13q deletion (23/32, 72%), and 14q deletion (16/32, 45%) (Fig. 5). A more focused analysis was performed to illustrate the potential discrepancies between RIGs (*n* = 32) and the non-RIG pedGBM_RTK1 tumors (*n* = 20) regarding their respective characteristic copy-number changes, with both groups exhibiting a similar pattern of chromosomal gains and losses (Suppl. Fig. 1a, c). The number of chromosomal breakpoints in RIGs and non-RIG pedGBM_RTK1 tumors were further visually determined to estimate the extent of genomic rearrangement caused by double-strand DNA breaks. Overall, RIGs display a significantly wider range and a higher number of chromosomal breakpoints (median: 38.5, range: 4–134) compared to non-RIG pedGBM_RTK1 tumors (median: 23, range: 13–51; two-sided *t*-test: *p* < 0.04) (Suppl. Fig. 1 d).

### Genomic profile of radiation-induced gliomas

RNA sequencing (*n* = 9), gene panel (*n* = 5), and whole-exome (*n* = 13) sequencing with matched blood samples were performed for a subset of RIG samples. None of the RIGs harbored somatic hotspot mutations in genes encoding histone 3 variants, *IDH1/2*, *BRAF*, or in the *TERT* promoter region. Four out of 18 tested samples (22%) exhibited somatic mutations in the *TP53* gene. Additional somatic mutations included *CBL*, *PDGFRA*, *NTRK2*, *EGFR*, *RAF1*, *ATRX*, and *BCOR* (Suppl. Table 1). One patient (RIG_25) harbored a *TP53* germline splice site mutation. One patient (RIG_14) was clinically diagnosed with Li-Fraumeni syndrome, but tumor material was insufficient for molecular testing. Further germline alterations associated with cancer predisposition syndromes were absent in the remainder of cases with germline material available (*n* = 13).

**Fig. 6:**
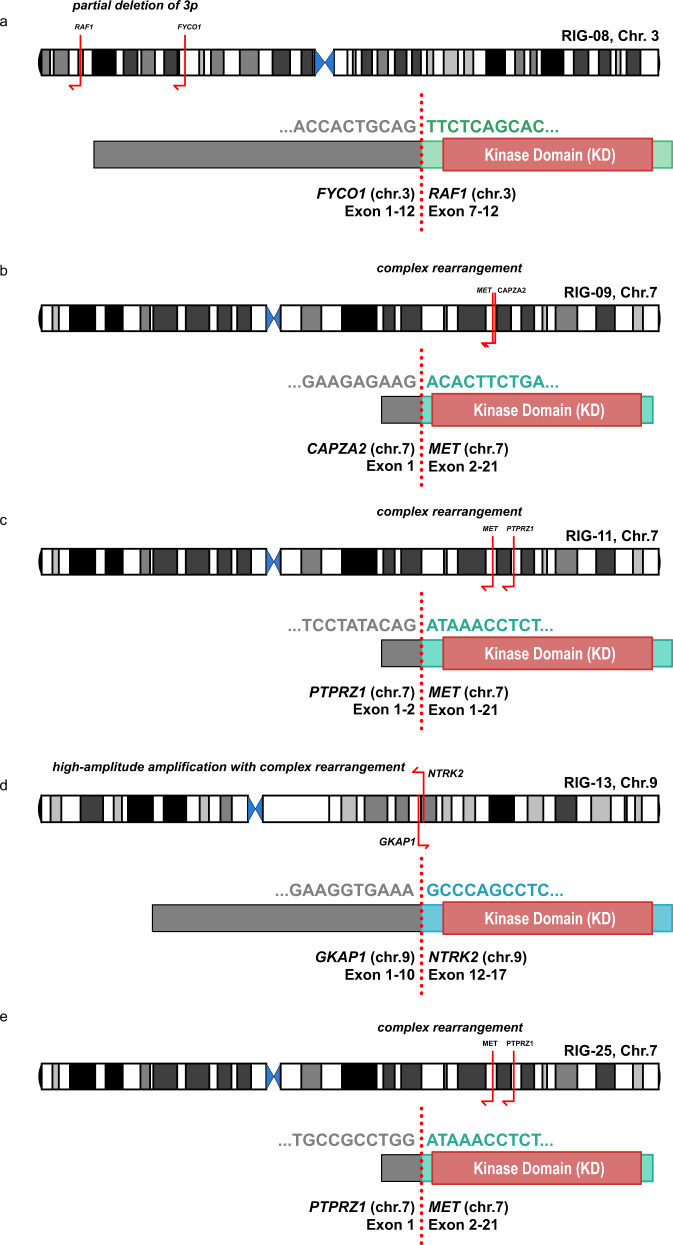
Alternative genetic rearrangements leading to aberrant MAPK/ERK pathway signaling. Partial deletions of chromosomal arm 3p induce formation of a *FYCO1:RAF1* fusion (**a**), with occurrence of a *GKAP1:NTRK2* fusion (**d**) emerging through inversion of a chromosomal arm 9q segment. Both rearrangements are predicted to lead to constitutive activation of the kinase domain (KD). Induced by complex rearrangements, *CAPZA2:MET* (**b**) and *PTPRZ1:MET* fusion (**c**, **e**) contained almost the full length MET protein, with overexpression driven by the highly active *CAPZA2* or *PTPRZ1* promoter.

Analysis of RNA sequencing data (*n* = 9) revealed relevant gene fusions including *PTPRZ1:MET* (RIG_11, RIG_25), *CAPZA2:MET* (RIG_09), *FYCO1:RAF1* (RIG_08), and *GFAP1:NTRK2* (RIG_13) (Fig. 6). Interestingly, the *FYCO1:RAF1* and *GKAP1:NTRK2* fusions were detected in two of the three tumors from the PXA-like subgroup based on DNA methylation profiling (Fig. 3). Both rearrangements are predicted to lead to a constitutive activation of the kinase domain, via loss of the N-terminal regulatory domain (RAF1) or constitutive dimerization (NTRK2). Deriving from a complex structural rearrangement, *PRPRZ1:MET* fusion proteins harbor an almost full length *MET* protein driven by the highly active *PTPRZ1* promoter. All *MET*-fused cases (RIG_09, RIG_11, RIG_25) demonstrated amplification of *MET* on copy-number profiles derived from DNA methylation arrays. While all four genetic rearrangements, which result in aberrant MAPK/ERK pathway activation, were reported in high-grade gliomas, none of them were previously discovered in RIGs^34–36^.

### Transcriptional profiling of radiation-induced gliomas

**Fig. 7:**
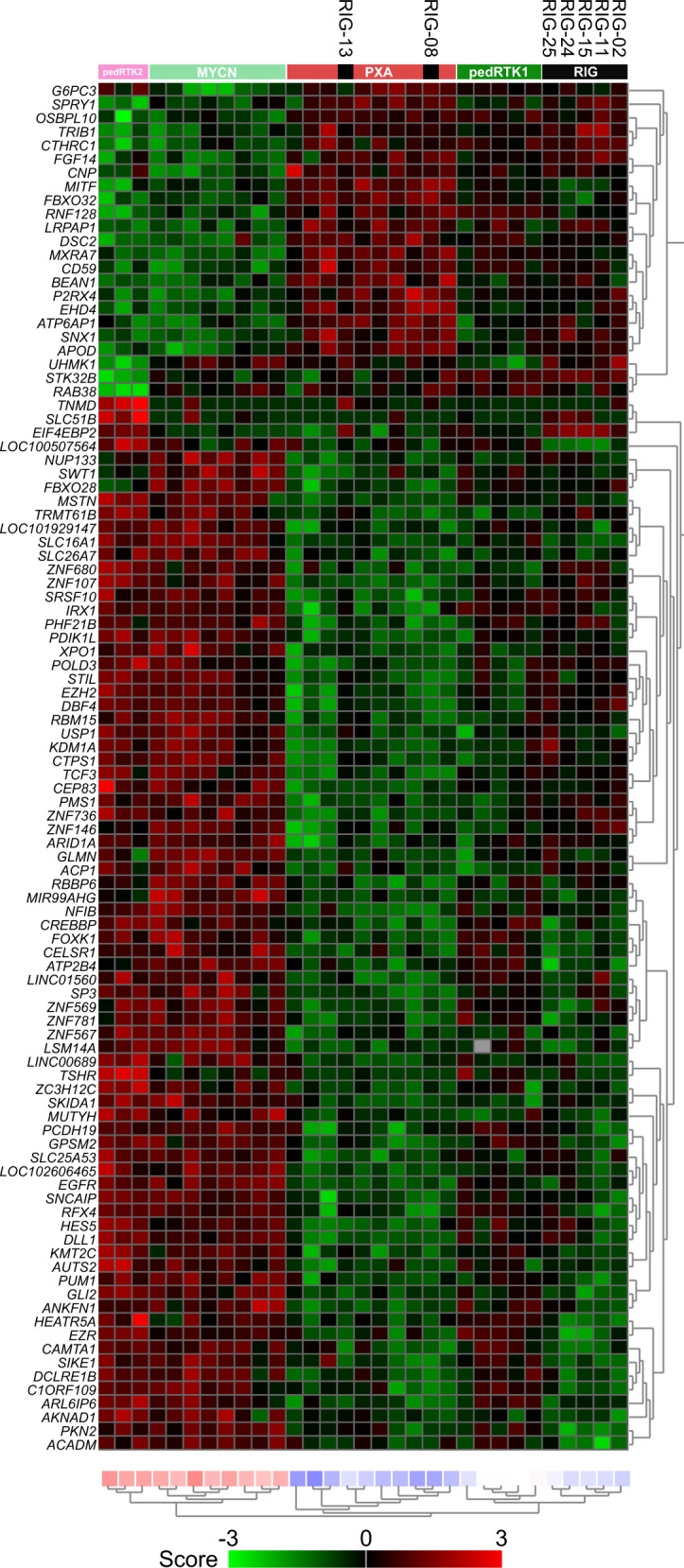
Heatmap representing the expression levels of the 100 most significant, differentially expressed genes comparing RIG samples with reference groups (PXA, pedGBM_RTK1, pedGBM_RTK2, and pedGBM_MYCN). Unsupervised hierarchical clustering reveals that gene expression profiling reflects our aforementioned findings based on DNA methylation profiling, with most RIGs resembling the pedGBM_RTK1 subgroup and a small subset more similar to PXAs. Each column represents one sample and each lane represents one gene. Expression levels are indicated by a color scale as indicated. PXA pleiomorphic xanthoastrocytoma, pedGBM_RTK1 pediatric glioblastoma subclass RTK1, pedGBM_RTK2 pediatric glioblastoma subclass RTK2, pedGBM_MYCN pediatric glioblastoma subclass MYCN.

Array-based gene expression analysis was performed on seven RIG samples as well as 24 additional tumors representing the H3/IDH-wildtype pediatric HGG pedGBM_RTK1 (*n* = 5), pedGBM_RTK2 (*n* = 3), pediatric HGG MYCN (*n* = 8)^32^, and PXA (*n* = 8) subgroups. Unsupervised hierarchical clustering analysis based on the 100 most differentially expressed genes recapitulated the methylation-defined subgrouping, with the largest fraction of the RIGs forming a homogenous expression pattern, resembling pedGBM_RTK1 tumors. Two cases (RIG_08, RIG_13), previously categorized into the PXA methylation group, also displayed an expression profile similar to PXAs (Fig. 7). The level of expression glioma-characteristic genes (e.g. CD34, GFAP, OLIG2, MAP2, MKI67, RBFOX3, SOX2, SYN; Suppl. Fig. 2) between RIGs and sporadic H3/IDH-wildtype pediatric HGGs did not reveal any notable differences, supporting the conclusion of a general similarity in their pathway activation.

## Discussion

RIGs represent a fatal long-term side effect of cranial irradiation^2,3,19,37–43^. A better understanding of the occurrence of these tumors and their molecular background is therefore of important clinical relevance. Our study supports the findings that RIGs harbor recurrent genetic alterations converging on an aberrant activation of the MAPK/ERK pathway (particularly via *PDGFRA*) together with loss of cell cycle control, facilitating tumorigenesis^7,20,35,36^. Importantly, we show that RIGs harbor a largely homogenous genetic and epigenetic profile, closely resembling sporadic pediatric GBM RTK1 tumors^32^. This suggests that both radiation-induced and sporadic pedGBM_RTK1 tumors might share a common cell of origin, which could be particularly vulnerable to ionizing radiation. However, the outcome of RIG patients is particularly poor, and worse than what has previously been described for sporadically occurring PDGFRA-amplified, 9p21-deleted pediatric GBM RTK1 tumors^32^. The discrepancy could suggest that additional oncogenic mechanisms might play a role during RIG formation, e.g. the trend towards greater genomic instability in the RIG cohort, as indicated by the elevated frequency of chromosomal breakpoints compared to their sporadic counterparts, potentially caused by radiation-induced DNA double-strand breaks. On the other hand, the RIG patient cohort also typically represents children with a long history of often aggressive prior treatment for their primary malignancy, which means that certain treatment options typically available at first diagnosis of GBM (e.g. radiotherapy) may no longer be an option for RIG patients^44,45^. It is also anticipated that the general health status of RIG patients is likely to be worse than that of primary-diagnosis counterparts. Further, some RIGs arising after MB were treated with an MB relapse protocol on the assumption that the new lesion was a recurrence of the primary tumor in the absence of biopsy confirmation, posing a possible risk that these protocols may be less efficacious against high-grade glioma than upfront GBM protocols. Thus, there are a number of key clinical reasons why an inferior survival might be expected, in addition to molecular mechanistic differences.

A small subset of RIGs with a more PXA-like profile harbored potentially druggable alterations in *RAF1* and *NTRK2*, and it will be of interest to investigate the precise characteristics of this group in larger cohorts. Our findings also do not at present suggest a substantial contribution of germline alterations in known tumor-associated genes in the occurrence of RIGs. However, further comprehensive germline analyses with matched blood samples are required to elucidate in full the potential role of hereditary predisposition in RIG formation.

RIG following MB of various subtypes as well as after ALL displayed converging fingerprints, despite the diversity of applied radiation protocols (MB-RIGs arose mostly at the site of local boost, while ALL-RIGs occurred throughout the cranial radiation field). All of the four main molecular MB subgroups were represented in the primary lesions, with no recurrent alterations observed in the primary MBs. Our findings unfortunately suggest that no conclusions can currently be drawn regarding predictive features in the patient or primary tumor for assessing the probability of subsequent RIG formation. An important question to address in future will be the precise role of radiotherapy protocols (craniospinal vs local boost, fractionation schema, photons/protons/heavy ions) in determining the risk of secondary malignancy^46–50^.

The ALL-RIGs included in our series were all diagnosed with their second tumor between 9 and 18 years of age, suggesting a particularly vulnerable but relatively narrow time frame for RIG occurrence after ALL. If confirmed in larger series, this could be an important variable for the planning of surveillance in this patient population. In contrast, both the age at diagnosis and latency after the primary tumor were much more varied for medulloblastoma. The earliest RIG occurred after a latency period of only 2 years following treatment for MB – within a timeframe which could easily allow misidentification as a recurrence of the primary tumor. Molecular characterization of 31 clinically-presumed relapse MBs within the prospective INFORM study^51^ revealed that 6/31 ‘relapsed’ MBs (19%) were in fact secondary HGG (unpublished data). In light of their aggressive clinical course and fundamentally different biology, it is essential to distinguish newly-arising RIGs from true recurrences in order to adjust treatment planning as early as possible to increase treatment efficacy. Thus, biopsy of recurrent lesions after MB is clearly warranted and should be considered whenever surgically feasible. Furthermore, the observation that a subset of prognostically favorable MBs (e.g. the MB-WNT subgroup) are also at risk of developing RIGs, highlights the urgency of current efforts to further examine the application of alternative treatment modalities (e.g. proton/heavy ion beam therapy) or the general de-intensification of radiation therapy in these patients^47,52–58^.

In summary, our findings demonstrate that RIGs are an aggressive, relatively homogenous group of CNS tumors with recurrent amplification of *PDGFRA* and loss of *CDKN2A/B* in the absence of somatic H3/IDH hotspot mutations. Our study uncovers possible similarities in origins with the pedGBM_RTK1 group of sporadic HGG, which will be important for further understanding the mechanisms by which these secondary tumors arise.

## Methods

### Patient population and tumor samples

Criteria for patient selection adapted from Cahan et al.^59,60^ included (1) a glioma emerging from the previously irradiated field, and (2) a non-glial primary malignancy, in this series MB (*n* = 23) and ALL (*n* = 9), to exclude potential tumor progression/recurrence not directly related to radiotherapy^61–63^.

This study is covered by the ethical approval of the Heidelberg University’s medical faculty. Additional clinical data and tumor material were collected by international collaborating centers according to local ethical and institutional review board approval and collected at the German Cancer Research Center (DKFZ, Heidelberg, Germany). Informed consent for molecular profiling was obtained from all patients and their legal representatives.

### DNA methylation profiling

The Illumina Infinium HumanMethylation450 (450k) array and Illumina Infinium MethylationEPIC (EPIC) array were used to obtain genome-wide DNA methylation profiles, according to the manufacturer’s instructions (Illumina, San Diego, USA). Data were generated at the Genomics and Proteomics Core Facility of the DKFZ (Heidelberg, Germany) and St. Jude Children’s Research Hospital (Memphis, USA). DNA methylation data was generated from both fresh-frozen and formalin-fixed paraffin-embedded (FFPE) tissue samples. On-chip quality metrics of all samples were carefully controlled.

Copy-number variation (CNV) analysis from 450k and EPIC methylation array data was performed using the conumee Bioconductor package version 1.12.0.

All computational analyses were performed in R version 3.4.4 (R Development Core Team, 2019). Raw signal intensities were obtained from IDAT-files using the minfi Bioconductor package version 1.24.0. Illumina EPIC and 450k samples were merged to a combined data set by selecting the intersection of probes present on both arrays (combineArrays function, minfi). Each sample was individually normalized by performing a background correction (shifting of the 5th percentile of negative control probe intensities to 0) and a dye-bias correction (scaling of the mean of normalization control probe intensities to 10,000) for both color channels. Subsequently, a correction for the type of material tissue (FFPE/frozen) and array (450k/EPIC) was performed by fitting univariate, linear models to the log2-transformed intensity values (removeBatchEffect function, limma package version 3.34.5). The methylated and unmethylated signals were corrected individually. Beta-values were calculated from the retransformed intensities using an offset of 100 (as recommended by Illumina).

Before further analysis, the following filtering criteria were applied: Removal of probes targeting the X and Y chromosomes (*n* = 11,551), removal of probes containing a single-nucleotide polymorphism (dbSNP132 Common) within five base pairs of and including the targeted CpG-site (*n* = 7998), probes not mapping uniquely to the human reference genome (hg19) allowing for one mismatch (*n* = 3965), and 450k array probes not included on the EPIC array. In total, 428,230 probes were kept for downstream analysis^64–66^.

To perform unsupervised dimension reduction, the remaining probes were used to calculate the 1-variance weighted Pearson correlation between the samples. The resulting distance matrix was used as input for t-SNE analysis (t-Distributed Stochastic Neighbor Embedding; Rtsne package version 0.13). The following non-default parameters were applied: theta = 0, pca = F, max_iter = 2500 perplexity = 20.

To perform unsupervised hierarchical clustering, the 10.000 probes with highest standard deviation were selected to calculate the Euclidean distance between samples, followed by applying Wards linkage method for sample clustering. In the heatmap, representation probes were reordered by complete linkage hierarchical clustering of the Euclidean distance between probes.

To evaluate focal amplifications and deletions and chromosomal gains and losses, we visually inspected copy-number profiles of each case. Candidate genes and their 3′ and 5′ intergenic neighborhood were further investigated using the Integrative Genomic Viewer (IGV) for the presence of breakpoints, as an indication for potential gene fusions.

### Gene expression profiling

Tumor samples with sufficient high-quality RNA were analyzed on the Affymetrix GeneChip Human Genome U133 Plus (v.2.0) Array (Affymetrix, Santa Clara, USA) at the Microarray Department of the University of Amsterdam, the Netherlands or the Genomics ad Proteomics Core Facility of the German Cancer Research Center (DKFZ). Subsequent library preparation, hybridization, and quality control was conducted following the manufacturer’s guideline. Expression data were normalized using the MAS5.0 algorithm. Identification of significantly differentially expressed genes between subgroups (test: anova; correction for multiple testing: false discovery rate, transform: *z*-score) and unsupervised hierarchical clustering were performed within the R2: Genomics Analysis and Visualization Platform (http://r2.amc.nl).

### Next-generation gene panel, whole-exome, low-coverage whole genome, and RNA sequencing

For the detection of single-nucleotide variations (SNVs), small insertions and deletions (indels), and gene fusions, a subset of tumor samples (*n* = 5) were analyzed via a customized enrichment/hybrid-capture-based next-generation sequencing gene panel^67^. DNA from FFPE tissue was extracted using the Promega Maxwell device (Promega), sheared on Covaris M220 (Covaris), according to the manufacturer’s guidelines. Following a successful quality control using a Bioanalyzer 2100 (Agilent), sequencing was performed on a NextSeq 500 instrument (Illumina). Paired-end sequencing was applied to increase the detection sensitivity of duplicates and possible gene fusions.

For whole exome (WES; *n* = 13) and RNA sequencing (*n* = 9), library preparation, sequencing and data processing were conducted following the pipeline established in the INFORM study^51^. In brief, library preparation for WES was performed using the Agilent SureSelectXT Human V5 kit. Prepared libraries were sequenced together with a tumor cDNA library (poly(A) + RNA, Illumina TruSeq RNA Kit v2) on an Illumina HiSeq. The 1000 Genomes phase 2 human reference assembly (NCBI build 37.1) was selected for mapping the sequencing reads using BWA (version 0.6.2). For detection of SNVs and indels, custom pipelines were used^68^. In brief, the computational analysis was performed using SAMtools mpileup and bctools version 0.1.19 to detect somatic variants. To annotate variants, ANNOVAR was applied with Gencode version 17. All high confidence coding or splice site germline variants in a selected panel of cancer predisposition genes were extracted using a custom Python script. To call short insertions or deletions (InDels) in tumor and control blood BAM files, the extracted data was annotated using Platypus (version 0.5.2/0.7.4 with parameters genInDels = 1, genSNPs = 0, ploidy = 2, nIndividuals = 2). Poor genotype quality and low variant counts in the tumor were excluded for the subsequent analysis. RNA sequencing data were analyzed with deFuse to detect gene fusions^69^.

Potentially relevant somatic and germline alterations were manually assessed and cross-examined through various databases (http://www.ncbi.nlm.nih.gov/SNP build 135, http://www.1000genomes.org, http://exac.broadinstitute.org, https://cancer.sanger.ac.uk/cosmic, https://www.ncbi.nlm.nih.gov/clinvar/).

### Statistics

Overall survival (OS) and progression-free survival (PFS) were analyzed by Kaplan–Meier analysis and tested for significant differences using a log-rank test. Binary and categorical patient characteristics between different subgroups were compared by a two-sided Fisher’s exact test. *P*-values < 0.05 were considered significant.

### Reporting summary

Further information on research design is available in the Nature Research Reporting Summary linked to this article.

## Supplementary information

Supplementary Information

Reporting Summary

## Data Availability

Methylation array data from this study have been deposited in GEO: GSE131482. Gene expression array were deposited in GEO: GSE168457. DNA sequencing data are available from the European Genome-Phenome Archive (EGA) under accession EGAS00001005243. Source data are provided with this paper.

## Source data

Source Data
