# Supplementary material for: Radiation-induced gliomas represent H3-/IDH-wild type pediatric gliomas with recurrent PDGFRA amplification and loss of CDKN2A/B
Source: Nat Commun. 2021 Sep 20;12:5530. doi: 10.1038/s41467-021-25708-y (PMC8452680; doi:10.1038/s41467-021-25708-y)
Supplement: Supplementary file 1 — Supplementary Information [file 41467_2021_25708_MOESM1_ESM.pdf]

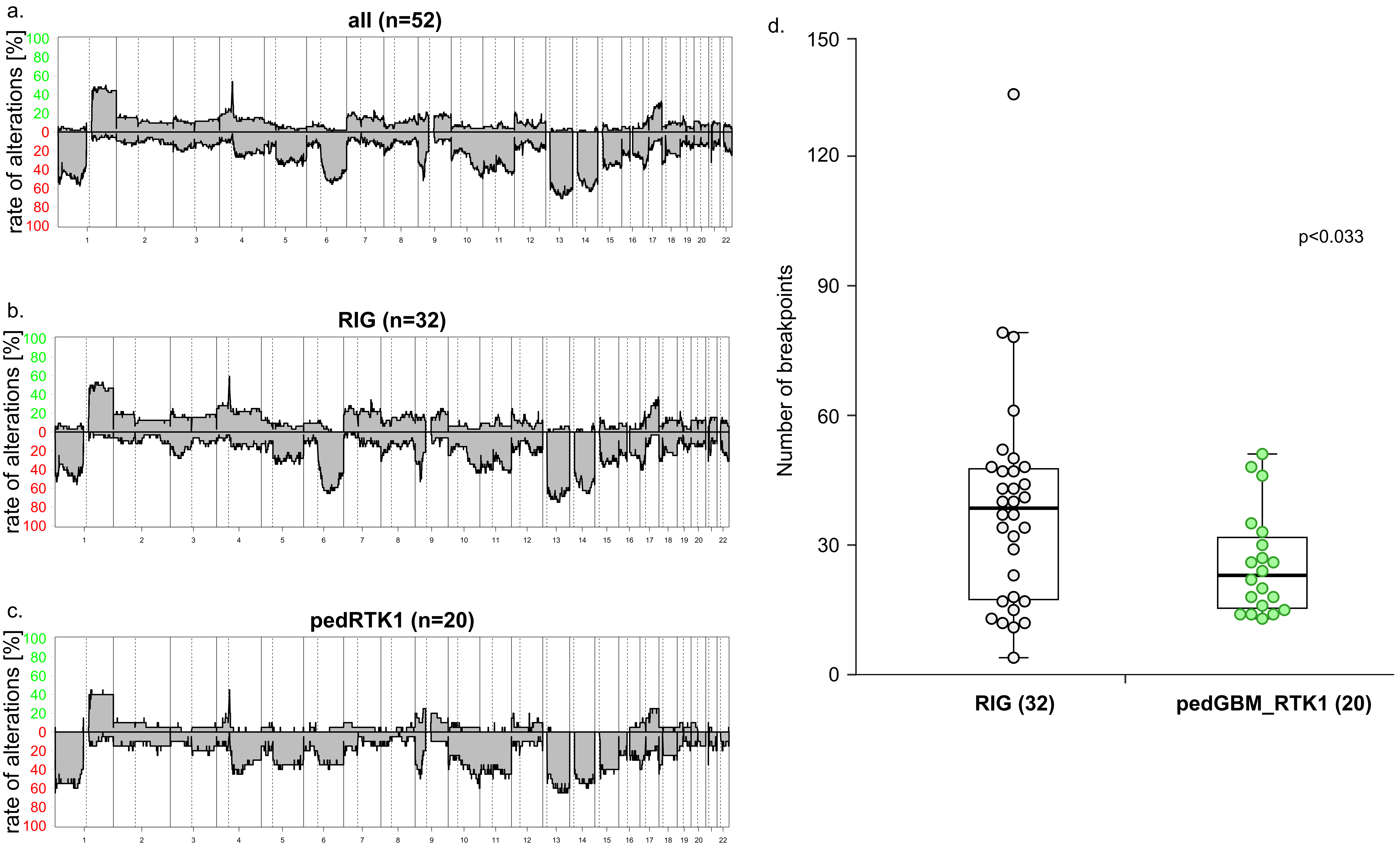

Supplementary Figure 1. Comparison of copy-number changes between RIGs (n=32) and non-RIG pedGBM_RTK1 tumors (n=20) reveal similar chromosomal gains and losses across all autosomal chromosomes (a-c). Number of chromosomal breakpoints in RIGs (n=32) and non-RIG pedGBM_RTK1 tumors (n=20) were manually counted through visual inspection of the single-case copy number plots (d). Data points are illustrated as boxplots with the median as middle line and bounds of boxes constraining the interquartile range. Lower and upper whiskers constrain data points between minimum and maximum. Outliers are depicted using individual data points beyond the whiskers. A statistical comparison between the RIG and non-RIG tumors was performed with a 2-sided t-test. If certain tumor samples display >10 breakpoints on a single chromosome, this particular chromosome received the value of 10 to avoid an exceedingly high overall number of breakpoints for the whole tumor. Overall, RIGs display a wider range and a significantly higher number of chromosomal breakpoints (median: 38.5, range: 4-134) compared to non-RIG pedGBM_RTK1 tumors (median: 23, range: 13-51; two-sided t-test: p=0.03319).

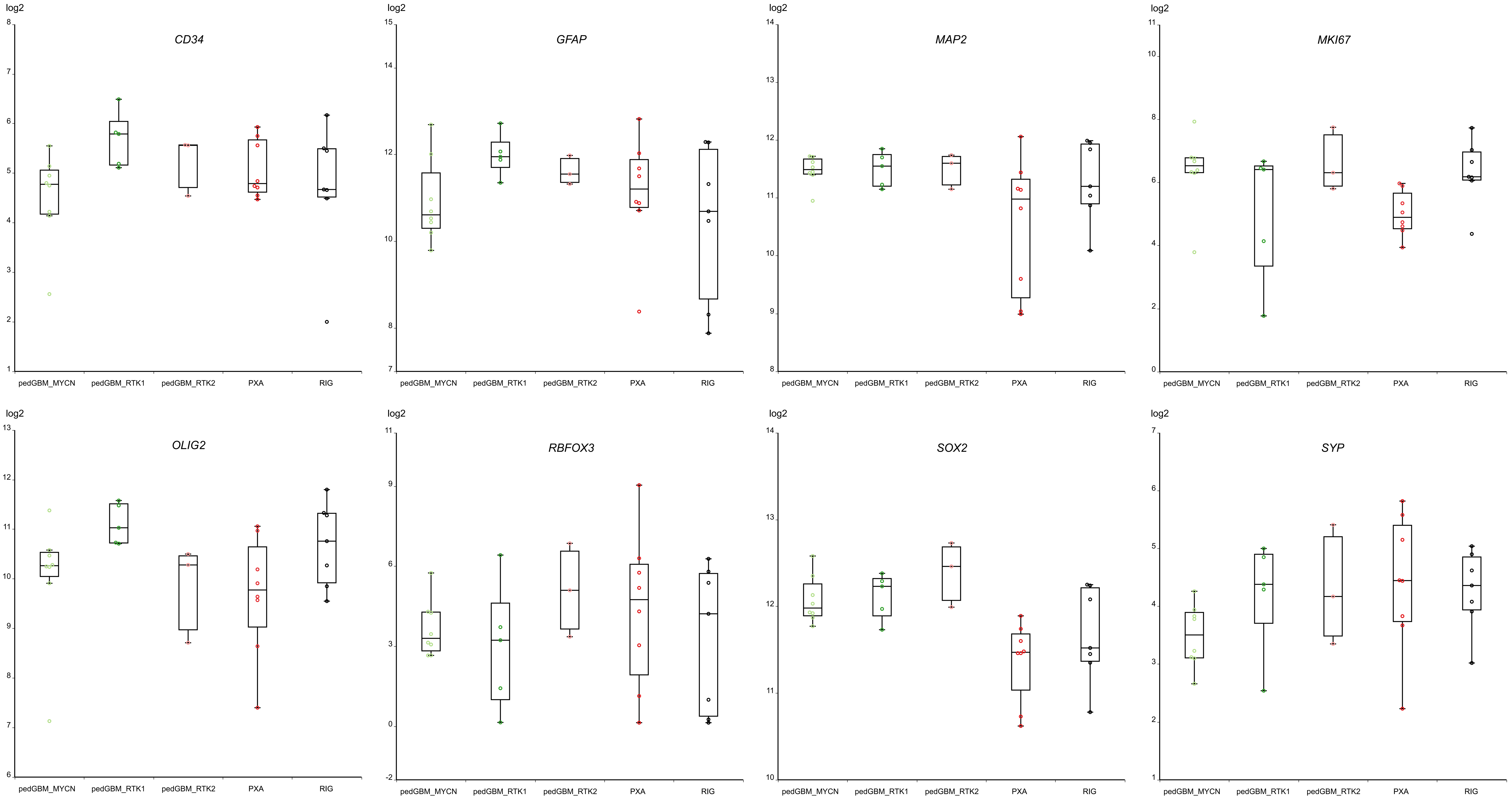

Supplementary Figure 2 Level of expression in glioma-characteristic genes comparing RIG samples (n=7) with pediatric glioblastoma subclass MYCN (pedGBM_MYCN, n=8), pediatric glioblastoma subclass RTK1 (pedGBM_RTK1, n=5), pediatric glioblastoma subclass RTK2 (pedGBM_RTK2, n=3) and pleiomorphic xanthoastrocytoma (PXA, n=8). Data are illustrated as boxplots with the middle lines as median and bounds of boxes illustrating the interquartile range. The x-axis illustrates the tumor entities and y-axis indicates expression levels logarithmically (log2). Lower and upper whiskers constrain data points between minimum and maximum. Outliers are depicted using individual data points beyond the whiskers. *OLIG2* Oligodendrocyte Transcription Factor 2, *GFAP* glial fibrillary acidic protein, *MAP2* Microtubule-associated protein 2, *MKI67* Marker of Proliferation Ki-67, *RBFOX3* RNA Binding Fox-1 Homolog 3, *SOX2* SRY-box transcription factor 2, *SYN* synapsin.

New_RIG_ID	Methylation subgroup	Location	Age category at diagnosis (RIG)	Gender (m/f)	Primary malignancy (type)	Latency (years)	death (y/n)	OS (months)	Methylation data available	Whole-exome data available	Gene panel data available	Matching blood sample available	RNA data available	Gene expression available	TP53 status	SETD2 status	Additional potentially relevant mutations
RIG_01	pedGBM_RTK1	frontal lobe	11-15	m	ALL	NA	y	10	x								
RIG_02	pedGBM_RTK1	left parietal	11-15	m	ALL	7	y	6	x	x		x	x	x	p.226_227del	wt	
RIG_03	pedGBM_RTK1	parietal lobe	11-15	f	ALL	10	y	3	x								
RIG_04	pedGBM_RTK1	parietal lobe	11-15	m	ALL	NA	NA	NA	x	x		x			wt	wt	CBL (p.Y371H)
RIG_05	pedGBM_RTK1	left frontal	11-15	f	ALL	4	NA	NA	x	x		x			p.R248W	wt	PDGFRA (p.L275S)
RIG_06	pedGBM_RTK1	frontal lobe	11-15	m	ALL	6	NA	NA	x								
RIG_07	pedGBM_RTK1	cerebellum	11-15	f	ALL	NA	y	8.5	x								
RIG_08	**PXA**	parietal left	6-10	f	ALL	3	NA	NA	x	x		x	x	x	wt	wt	
RIG_09	pedGBM_RTK1	cerebellar	11-15	f	MB Group4	4	y	8	x	x		x			wt	p.S1002fs	
RIG_10	pedGBM_RTK1	cerebellum	6-10	m	MB Group3	3	y	3	x	x	x	x	x	x	wt	wt	NTRK2 (p.L806F)
RIG_11	pedGBM_RTK1	cerebellum	11-15	m	MB Group4	3	NA	NA	x								
RIG_12	**PXA**	temporal	>21	m	MB	13	y	10	x	x		x	x	x	p.G154fs	wt	
RIG_13	pedGBM_RTK1	NA	11-15	m	MB SHH-AD	4	y	11	x								
RIG_14	pedGBM_RTK1	cerebellum	>21	m	MB	NA	n	18	x	x		x	x	x	wt	wt	PDGFRA (p.D143N)
RIG_15	pedGBM_RTK1	cerebellum	16-20	m	MB	9	NA	NA	x								
RIG_16	pedGBM_RTK1	cerebellum	0-5	m	MB	1.5	NA	NA	x								
RIG_17	pedGBM_RTK1	cerebellum	>21	f	MB	10	NA	NA	x		x				wt	wt	EGFR (p.D1051H)
RIG_18	pedGBM_RTK1	parieto-occipital	11-15	f	MB	NA	NA	NA	x	x		x			wt	wt	
RIG_19	pedGBM_RTK1	cerebellum	11-15	f	MB	6	NA	NA	x								
RIG_20	**PXA**	cerebellum	11-15	m	MB	3	y	5	x								
RIG_21	**PXA**	cerebellum	11-15	f	MB	NA	NA	NA	x								
RIG_22	pedGBM_RTK1	cerebellum	0-5	f	MB	NA	NA	NA	x								
RIG_23	pedGBM_RTK1	cerebellum	>21	m	MB	14	y	9	x	x		x	x		wt	p.N1396fs	RAF1 (p.S246A)
RIG_24	pedGBM_RTK1	frontal lobe	>21	f	MB	15	y	2	x	x		x	x	x	wt	p.G720fs	NF1 (p.W1931X)
RIG_25	pedGBM_RTK1	cerebellum	16-20	f	MB	4	y	4	x	x		x	x	x	p.A276G, c.97-1G>T (germline)	wt	ATRX (p.S1108X)
RIG_26	pedGBM_RTK1	cerebellum	>21	m	MB	NA	NA	NA	x								
RIG_27	pedGBM_RTK1	cerebellum	6-10	m	MB Group3	6	y	9	x		x				wt	wt	BCOR (p.P670A)
RIG_28	pedGBM_RTK1	cerebellum	>21	m	MB Group4	9	NA	NA	x								
RIG_29	pedGBM_RTK1	cerebellum	6-10	f	MB SHH-INF	4	y	8	x		x				wt	wt	
RIG_30	pedGBM_RTK1	cerebellum	11-15	f	MB SHH-AD	2.5	y	11	x						wt	wt	
RIG_31	pedGBM_RTK1	cerebellum	11-15	f	MB Group4	5	n	3	x		x				wt	wt	
RIG_32	pedGBM_RTK1	cerebellum	16-20	m	MB WNT	3	y	15	x								
RIG_33	pedGBM_RTK1	cerebellum	>21	m	MB Group4	10	y	7	x								

Supplementary Table 1. Summary of clinical patient data, TP53- and SETD2-status. MB medulloblastoma, MB-WNT medulloblastoma WNT subgroup, MB-SHH-INF medulloblastoma sonic-hedgehog infant subgroup, MB-SHH-AD medulloblastoma sonic-hedgehog adult subgroup, PXA pleiomorphic xanthoastrocytoma, pedGBM_RTK1 pediatric glioblastoma subclass RTK1, NA not applicable, OS overall survival, wt wildtype.

RIG_ID	EGA-ID
RIG_02	INF_R_148
RIG_08	INF_R_490
RIG_09	INF_R_1540
RIG_11	INF_P_51
RIG_13	INF_R_013
RIG_15	INF_R_118
RIG_23	INF_R_223
RIG_24	INF_R_212
RIG_25	INF_R_431

Supplementary Table 2. Matching RIG_XX to the EGA-IDs for the INFORM project INF_X_XXX (EGAS00001005243).
